# Influence of Quadrato Motor Training on Salivary proNGF and proBDNF

**DOI:** 10.3389/fnins.2019.00058

**Published:** 2019-02-07

**Authors:** Micaela Caserta, Tal D. Ben-Soussan, Valerio Vetriani, Sabrina Venditti, Loredana Verdone

**Affiliations:** ^1^National Research Council, Institute of Molecular Biology and Pathology, Rome, Italy; ^2^Cognitive Neurophysiology Laboratory, Research Institute for Neuroscience, Education and Didactics, Patrizio Paoletti Foundation for Development and Communication, Assisi, Italy; ^3^Department of Biology and Biotechnology “Charles Darwin”, Sapienza University of Rome, Rome, Italy

**Keywords:** Quadrato Motor Training, proNGF, proBDNF, neuroplasticity, neurotrophins, well-being

## Abstract

Previous studies demonstrated exercise-induced modulation of neurotrophins, such as Nerve Growth Factor (NGF) and Brain-Derived Neurotrophic Factor (BDNF). Yet, no study that we are aware of has examined their change as a function of different training paradigms. In addition, the understanding of the possible training-induced relationship between NGF and BDNF change is still lacking. Consequently, in the current study we examined the effect of a Walking Training (WT) and of Quadrato Motor Training (QMT) on NGF and BDNF precursors (proNGF and proBDNF). QMT is a specifically structured sensorimotor training that involves sequences of movements based on verbal commands, that was previously reported to improve spatial cognition, reflectivity, creativity as well as emotion regulation and general self-efficacy. In addition, QMT was reported to induce electrophysiological and morphological changes, suggesting stimulation of neuroplasticity processes. In two previous independent studies we reported QMT-induced changes in the salivary proNGF and proBDNF levels. Our present results demonstrate that following 12 weeks of daily QMT practice, proNGF level increases while proBDNF showed no significant change. More importantly, while no correlation between the two neurotrophins prior to training was detectable, there was a significant correlation between change in proNGF and proBDNF levels. Taken together the current results suggest that the two neurotrophins undergo a complex modulation, likely related to the different pathways by which they are produced and regulated. Since variations of these neurotrophins have been previously linked to depression, stress and anxiety, the current study may have practical implications and aid in understanding the possible physiological mechanisms that mediate improved well-being, and the dynamic change of neurotrophins as a result of training.

## Introduction

The understanding of the relationship between a healthy body and mind is of crucial relevance, but the physiological mechanisms underlying this relationship are still far from being clarified. Many studies have addressed this topic, highlighting that physical exercise can contribute to improved cognition and well-being, by directly affecting neuroplasticity (for a review see Voss et al., 2013b; Budde et al., 2016). Neuroplasticity is mediated by neurotrophic factors, among which Nerve Growth Factor (NGF) and Brain-Derived Neurotrophic Factor (BDNF). Neurotrophins are involved in the regulation of synaptic connectivity, fiber guidance and dendritic morphology in the peripheral and central nervous system (Bibel and Barde, 2000).

These factors are synthesized as precursors, proNGF and proBDNF, and released in the synaptic space, where they undergo cleavage and maturation, following which the mature forms are internalized via the binding to specialized receptors (Chao, 2003; Lu et al., 2005). Several studies have also underlined a relevant role for the precursor proteins in mediating axonal development and synaptic plasticity (Costa et al., 2018). Different types of environmental enrichment, many of which are based on physical activity and exercise, were found to be associated with modulation of proBDNF (Neeper et al., 1995; Ploughman, 2008; Rasmussen et al., 2009; Baroncelli et al., 2010; Sigwalt et al., 2011; Voss et al., 2013a,b). Research related to training-induced dependent changes of neurotrophins is not abundant (Neeper et al., 1996; Hong et al., 2015; Okudan and Belviranli, 2017; Arvidsson et al., 2018).

Very few studies, mostly conducted in animals, examined exercise-related proNGF changes (Chae et al., 2014; Ando et al., 2016). A rare example in human are the studies on the Quadrato Motor Training (QMT), a structured sensorimotor training, that combines motor and cognitive functions, such as reflectivity and spatial cognition (Dotan Ben-Soussan et al., 2013; Ben-Soussan et al., 2014) as well as emotional well-being (Paoletti et al., 2017; Piervincenzi et al., 2017). In addition, proNGF salivary levels of adults and children were found to decrease following 4 weeks of practice. Interestingly, decreased proNGF correlated with increased ideational flexibility and creativity (Venditti et al., 2015). To the best of our knowledge QMT is the only paradigm, so far investigated in humans, for which change in the level of NGF was observed. Moreover, proBDNF salivary levels were shown to increase following 12 weeks of daily QMT. Parallel MRI examination of the subjects involved in this study, revealed significant correlation of higher proBDNF levels with increased functional connectivity and neuronal arborization (Ben-Soussan et al., 2015b).

Nonetheless, to date, a unified picture of the effects of QMT on both proNGF and proBDNF is still lacking. Consequently, in the present work, we set up a more comprehensive study, in which we conducted the molecular analysis of both proNGF and proBDNF in the same group of subjects, before and after 12 weeks of practice. The aim of the current study is to improve the understanding of neurotrophins modulation induced by QMT compared to a simple walking training (WT), and to infer the possible different involvement of the two neurotrophins in response to practice.

## Materials and Methods

### Participants and Procedure

A total of 40 right handed female participants were enrolled in the study. All were healthy with no emotional or behavioral disorders, general cognitive disorders, or developmental coordination disorders or medical history that might affect their performance. This study was approved by the CNR Research Ethics and Bioethics Advisory Committee, Protocol AMMCNT-CNR n. 0080953, November 26th, 2015.

In the first visit the participants were introduced to the entire procedure, adequate understanding was tested, and the informed written consent was obtained, in accordance to the Declaration of Helsinki. The participants were randomly allocated to either the QMT or the control WT groups. The subjects were then explained the QMT or WT procedure and were first asked to donate a sample of saliva in triplicate for molecular assessments. They were also informed of the option of interrupting the training and dropping-out from the study at any time for any reasons, including changes of the clinical status that would impede continuation, refusal to continue and personal needs. The subsequent sessions of QMT or WT, were conducted at home. To check with the compliance to the exercise, subjects were asked to fill up a personal diary on daily bases to collect information about their practice and habits during the period of exercise. The collection of saliva samples was repeated in the laboratory after 12 weeks of daily training. Although the initial group size was identical (*n* = 20), the final number of participants finishing the 12 weeks of training was *n* = 13 for QMT and *n* = 11 for WT. Thus, the analyses were conducted on a total of 24 subjects finishing the 12 weeks training (mean age ± SD: 48.5 ± 10.6).

### Training Groups

#### Quadrato Motor Training (QMT)

**FIGURE 1:**
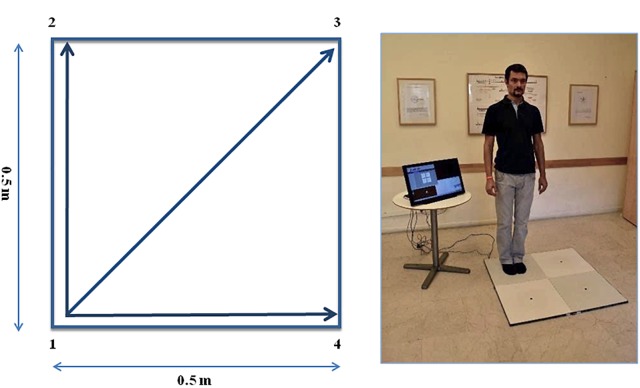
The Quadrato Motor Training (QMT). **(A)** A graphical illustration of the QMT. **(B)** A participant to a QMT session. Written informed consent was obtained for the publication of this image from the person in the picture, who has not taken part in the current research. Adapted from Ben-Soussan et al. (2014).

The QMT requires standing at one corner of 0.5 × 0.5 m square and making movements in response to a verbal command given by an audio recording. In the QMT space there are three optional directions of movement (Figure 1). At each corner there are three possible directions of movement, thus the training consists of 12 possible movements (3 directions × 4 corners). The entire protocol consists of 7 sequences, lasting 12 min. We used a movement sequence paced at a rate of an average of 0.5 Hz, comparable to a slow walking rate. For additional details see Dotan Ben-Soussan et al. (2013), Venditti et al. (2015).

#### Walking Training (WT)

The WT group was instructed to make successive steps following the auditory stimulus, keeping the same pace, duration of steps and auditory cue as the QMT, but the movement had to be free in the room space and not within the square. This group was, therefore, told to simply make the first step and then continue in response to the instructions, regardless the number specified by the recording. This reduced the uncertainty regarding the direction of the movement compared to the QMT group. The WT group was not informed about the QMT option relating the numbers to a specific location in the Quadrato space and have thus provided a control performing a task with similar motor demands, but with reduced cognitive ones.

### Molecular Examination

Analysis of salivary neurotrophins is a reliable non-invasive procedure (Lee and Wong, 2009; Jang et al., 2011; Jasim et al., 2018). The choice of saliva was taken because several studies reported that neurotrophins have widespread functions in the organism (Rothman et al., 2012; Marosi and Mattson, 2014), that are coordinated by an active communication between brain and periphery. In most instances molecular analysis of brain markers was shown to be conducted from saliva with good reliability (Nohesara et al., 2011; Smith et al., 2015).

### Saliva Samples Collection

Salivary proNGF and proBDNF were examined in triplicate to take in consideration the potential variability due to flow rate. Saliva samples were collected at day 1 and after 12 weeks in the morning between 10 and 11 am, and specific instructions were given to the participants including: avoid brushing teeth, using salivary stimulants and consuming a major meal within 1 h prior to collection, avoid consuming acidic or high sugar foods 20 min prior to collection. 10 min before collection the subject was suggested to rinse the mouth with water. Unstimulated whole saliva was collected by passive drool and stored at -80°C. Prior to electrophoresis the samples were subjected to vortex for 30 s and centrifuged at maximum speed for 15 min. Saliva supernatants were transferred to fresh tubes, a protease inhibitor cocktail was added (Roche, 04693116001) and total protein concentration was determined by Bradford assay (BIO-RAD).

### Western Blot Analysis

To evaluate the neurotrophin levels in the saliva sample of the participants before and after training, 15 μg of total proteins were subjected to electrophoresis on SDS–PAGE, (4–15% precast gradient gels) under semi-denaturing conditions: samples were loaded on the gels without the previous canonical pre-heating step. Under these conditions, we obtained a better resolution of the neurotrophin protein bands following hybridization with the specific antibody, avoiding interference with the highly concentrated amylase family proteins (Supplementary Figure S1). Following electrophoresis, the proteins were transferred onto 0.2 mm PVDF membranes by *Trans*-Blot Turbo Blotting System (BIO-RAD) and hybridized with appropriate amounts of anti-NGF and anti-BDNF antibodies. Antibody anti-NGF: SIGMA-ALDRICH, polyclonal N6655, dilution 1:4000, recognizes both pro and mature NGF (Soligo et al., 2015). Antibody anti-BDNF: Thermo Fisher, oligoclonal 710306, dilution 1:4000, recognizes both pro and mature BDNF. Incubation with both antibodies was followed by anti-rabbit secondary antibody treatment (Jackson ImmunoResearch, 111-035-003, dilution 1:20000).

### Quantification and Statistics

The whole family of bands corresponding to proBDNF and proNGF (MW: 50–100 KDa) was quantified relative to the total protein present on the membranes using Image Lab software according to manufacturer instructions. To answer the question regarding the effects of QMT on neurotrophic factors, we ran a Group (QMT, WT) × Training (pre-, post-) analysis of variance (ANOVA) separately for proNGF and proBDNF. We also ran a one-way analysis of covariance (ANCOVA). Then, the Pearson correlation between change in proNGF and proBDNF, was computed. Change in salivary neurotrophins was calculated by dividing their post-value by their pre-value.

## Results

### proNGF Levels Following 12 Weeks of QMT

**FIGURE 2:**
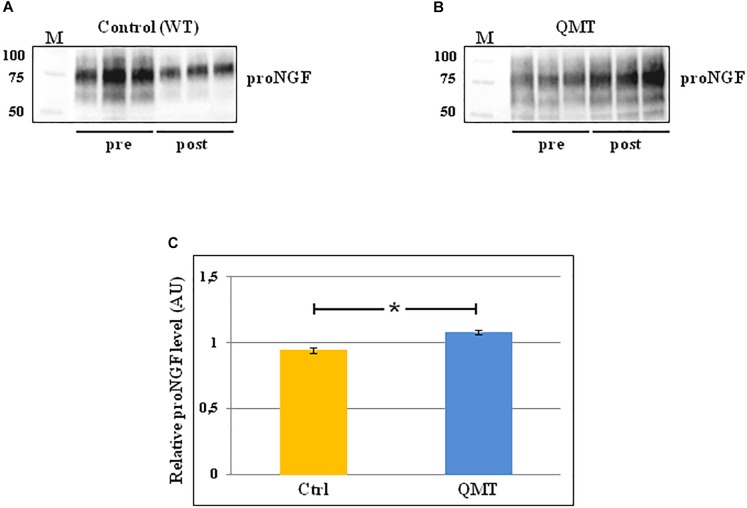
Western blot analysis of changes in proNGF levels for QMT and WT groups. **(A)** Representative gel for one WT participant. **(B)** Representative gel for one QMT participant. **(C)** The histograms show the post/pre-ratio of the proNGF mean log values of all the participants for both groups. Error bars indicate the SEM. ^∗^*p* < 0.05. M, Molecular Weight marker.

In order to evaluate the levels of proNGF in the saliva samples of the participants before and after 12 weeks of training, we used the western blot technique. The results of this analysis are shown in Figure 2. In the panels A and B of Figure 2 the representative gels for two participants, one control and one QMT, respectively, are illustrated. When looking at these two subjects, it appears that the levels of proNGF increase in the QMT samples after the training (QMT post; Figure 2, panel B), while the control samples shows a decrease (control post; Figure 2, panel A). As seen in panel A and B, proNGF is not present as a single molecular species, but as a family of bands, ranging from 50 to 100 KDa. This is due to multiple and variegated post-translational modifications, mainly glycosylation, that contribute to the heterogeneity of the molecular weights (Reinshagen et al., 2000). Because they represent different forms of proNGF, all the bands need to be necessarily taken into account when performing the quantification for each single subject. The quantitative analysis of proNGF levels was carried out for all subjects. Given non-normality of the data, these were log-transformed. The histograms in the panel C of Figure 2 show the post/pre-ratio of the mean values for both groups, indicating a proNGF increase in the QMT group (see also the change in proNGF values for the two separate groups in Supplementary Figure S2).

To answer the question regarding the effect of QMT on proNGF, we ran a Group (QMT, WT) × Training (pre, post) analysis of variance (ANOVA). A significant Group × Training interaction was found for proNGF [*F*(1,22) = 6.034, *p* = 0.022]. The effect size using partial η^2^ was 0.215.

We then supplemented our analysis with a one-way analysis of covariance (ANCOVA), with pre-QMT proNGF as the covariate, and post-QMT proNGF as the dependent variable. The main effect for Group was significant [*F*(1,21) = 6.98, MSE = 0.021, *p* = 0.015], with a significant proNGF post/pre-ratio increase for QMT compared to the WT group [*t*(22) = -2.69, *p* < 0.05] (see Figure 2, panel C).

Taken together, these results indicate that the practice of QMT for 12 weeks significantly increases proNGF, while this is not true for the WT group.

### proBDNF Levels Following 12 Weeks of QMT

**FIGURE 3:**
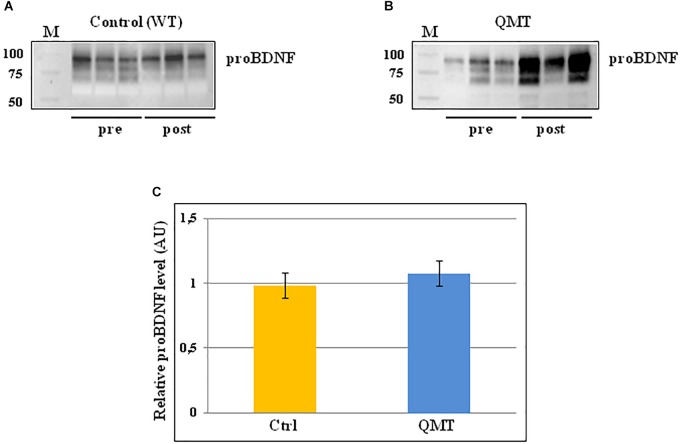
Western blot analysis of changes in proBDNF levels for QMT and WT groups. **(A)** Representative gel for one WT participants. **(B)** Representative gel for one QMT participant. **(C)** The histograms show the post/pre-ratio of the proBDNF mean log values of all the participants for both groups. Error bars indicate the SEM. ns, not significant. M, Molecular Weight marker.

The same analysis conducted for proNGF by western blot, was also carried out for proBDNF, and the results are shown in Figure 3. In the panels A and B of Figure 3, two representative gels for proBDNF are displayed, from one control and one QMT subject, respectively. As observed previously for proNGF, the proBDNF profile of the bands is complex, since also this neurotrophin undergoes multiple types of post-translational modifications (Smith et al., 2015). Therefore, even in this case the quantification was done taking into account the whole family of bands. The image shows that the QMT participant presents a higher level of salivary proBDNF following training (Figure 3, panel B) compared to the control (Figure 3, panel A). The quantitative analysis of proBDNF levels was carried out for all subjects. Given non-normality of the data, these were also log-transformed. The histograms in panel C of Figure 3 show the post/pre-ratio of the mean values for both groups (see also the change in proBDNF values for the two separate groups in Supplementary Figure S3).

When conducting the ANOVA, no significant main effects or interaction were found [*F*(1,22) = 0.19, 3.38 *ns*]. Similarly, the main effect for Group in the ANCOVA was also not significant [*F*(1,21) = 2.46, *MSE* = 0.005, *ns*].

### Correlation Between proNGF and proBDNF

**FIGURE 4:**
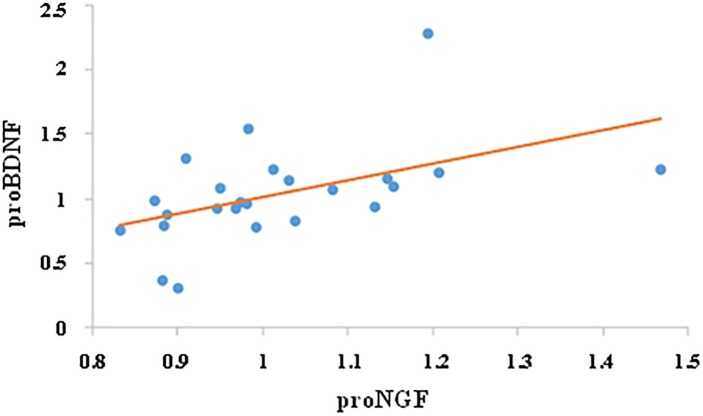
Correlation between proNGF and proBDNF. Change in salivary neurotrophins was calculated by dividing their post-value by their pre-value. Change in proNGF was significantly and positively correlated with change in proBDNF (*r* = 0.49, *p* < 0.05, *n* = 24).

We then investigated whether the change in proNGF level was correlated with change in proBDNF. While there was no correlation between proNGF and proBDNF levels prior to training (*r* = 0.28, ns), change in proNGF was significantly and positively correlated with change in proBDNF (*r* = 0.49, *p* < 0.05, *n* = 24, see Figure 4).

## Discussion

Neurotrophins play a key role in the central and peripheral nervous system, and drive neuroplasticity also during adult life. Neuroplasticity is a complex process that can be enhanced by several types of environmental enrichment, including physical and cognitive training, as well as learning-stimulating activities (Ploughman, 2008; Baroncelli et al., 2010; Voss et al., 2013a,b). While a large body of evidences have shown changes of proBDNF levels during training-induced neuroplasticity (Neeper et al., 1995; Rasmussen et al., 2009; Knaepen et al., 2010; Zoladz and Pilc, 2010), information regarding the involvement of proNGF in this process is largely lacking, with only few reports mostly on animal models (Chae et al., 2014; Ando et al., 2016).

In the present study we investigated the modulation of the salivary proNGF and proBDNF levels following 12 weeks comparing two types of training, namely QMT and WT. The main result obtained is increased proNGF following 12 weeks of QMT practice compared to the control group (Figure 2). The effect size was 0.215, which is considered a relatively moderate effect. While our previous work reported decreased proNGF after 4 weeks of QMT daily practice (Venditti et al., 2015), the current study suggests that performing the practice for 8 more weeks leads to a different outcome. Our hypothesis is that the proNGF decrease we previously detected after 4 weeks (Venditti et al., 2015), could be due to its fast utilization related to enhanced neuroplasticity, as supported by animal model studies (Bruno and Cuello, 2006). In turn, proNGF consumption stimulates further re-synthesis in the subsequent 8 weeks (Figure 2). The combination of the motor and cognitive elements in the QMT requires and reinforces dividing attention, and stimulates neuroplasiticty (Dotan Ben-Soussan et al., 2013; Ben-Soussan et al., 2015a; Lasaponara et al., 2017; Piervincenzi et al., 2017). The present results underline that it is possibly the cognitive component, absent in the WT, that allows QMT to stimulate the increase of proNGF levels. Although a trend to proBDNF enhancement was observed for the QMT group after 12 weeks of training (Supplementary Figure S3), it did not reach significance.

### Distinct Mechanisms of Change for proNGF and proBDNF

The apparently different behavior of the two molecules could be attributed to the distinct pathways by which they are synthesized and regulated (Mowla et al., 1999). In fact, proNGF is released through both the constitutive and regulatory secretory pathways in cells from peripheral tissues and nerves (Mowla et al., 1999; Costa et al., 2018), while proBDNF follows a regulated pathway that drives its synthesis upon stimulation of neuronal activity (Lu et al., 2005).

We argue that the molecular mechanisms underlying the variation in neurotrophins secretion, induced by QMT practice, are different for the two molecules analyzed. On one hand, proNGF could decrease during the first 4 weeks of training because of enhanced processing of the protein, followed by its transcriptional increase during the subsequent 8 weeks. On the other hand, the proBDNF trend to increase could be due to different regulatory pathways and/or different timing.

### Correlation Between Change in proNGF and proBDNF

Most of the evidences on exercise induced modulation of neurotrophins are related to BDNF analysis (for review see Neeper et al., 1995; Rasmussen et al., 2009; Voss et al., 2013b), a few are related to NGF (Chae et al., 2014; Ando et al., 2016), while works describing the relationship between the two are scarce. In fact, several papers have reported concomitant detection of BDNF and NGF levels changes following exercise (Neeper et al., 1996; Hong et al., 2015; Okudan and Belviranli, 2017; Arvidsson et al., 2018), without addressing the correlation between the two. Only one correlation study, outside the exercise field, was reported in rats, regarding the nervous system development, in which BDNF and NGF show reciprocal behavior during development, but parallel increase in the adult brain (Maisonpierre et al., 1990). Consequently, to better understand the possible relationship between these two neurotrophins, we examined the correlation between their changes following training.

While there was no correlation between proNGF and proBDNF before the training, a significant positive correlation was found between change in proNGF and proBDNF. One could hypothesize that the modulation of proNGF induces the variations of proBDNF or, alternatively, that the proBDNF increase stimulates the subsequent resynthesis of proNGF, though this has to be further examined.

### Limitations of the Study

There are a few limitations to the study that should be noticed. The first is the small sample size, only 13 and 11 participants for the QMT and control groups, respectively. The second is that the participants performed the training at home, and we checked for the exercise performance only at the end of the experiment, by looking at the daily calendar they had to tick. In the future, a study with a larger sample size should be conducted to extend the present results, using a more efficient method monitoring compliance to the practice (such as video camera recording).

To be able to distinguish between the relative contribution of proNGF and proBDNF to either the cognitive/attentive or the motor components of QMT, it will be important, in addition to the WT group, to introduce a non-practicing control group.

## Conclusion

The present study increases the current knowledge and understanding related to the involvement of proNGF and proBNDF in the neuroplasticity process induced by training and shows that 12 weeks of daily QMT practice increased proNGF, in contrast to a simple WT. This study highlights the relevance of analyzing molecular parameters, such as neurotrophins, and the dynamic/mutual change in their levels as a result of different motor training paradigms.

## Author Contributions

The entire project was designed through the concerted contribution of TB-S, LV, MC, and SV. LV, MC, SV, and VV performed the recruiting of the participants and the saliva sample collection. TB-S instructed the participants about the trainings and performed the statistical analysis of data. MC, LV, and VV performed most of the western blot analysis. LV did the quantification of results. SV contributed to the western blot analysis, participated in the interpretation of the results and wrote the manuscript. LV, MC, SV, VV, and TB-S theoretically contributed to the interpretation of the results and critically revised the manuscript.

## Conflict of Interest Statement

The authors declare that the research was conducted in the absence of any commercial or financial relationships that could be construed as a potential conflict of interest.
