# Supplementary figures and images for: Influence of Quadrato Motor Training on Salivary proNGF and proBDNF
Source: Front Neurosci. 2019 Feb 7;13:58. doi: 10.3389/fnins.2019.00058 (PMC6374314; doi:10.3389/fnins.2019.00058)

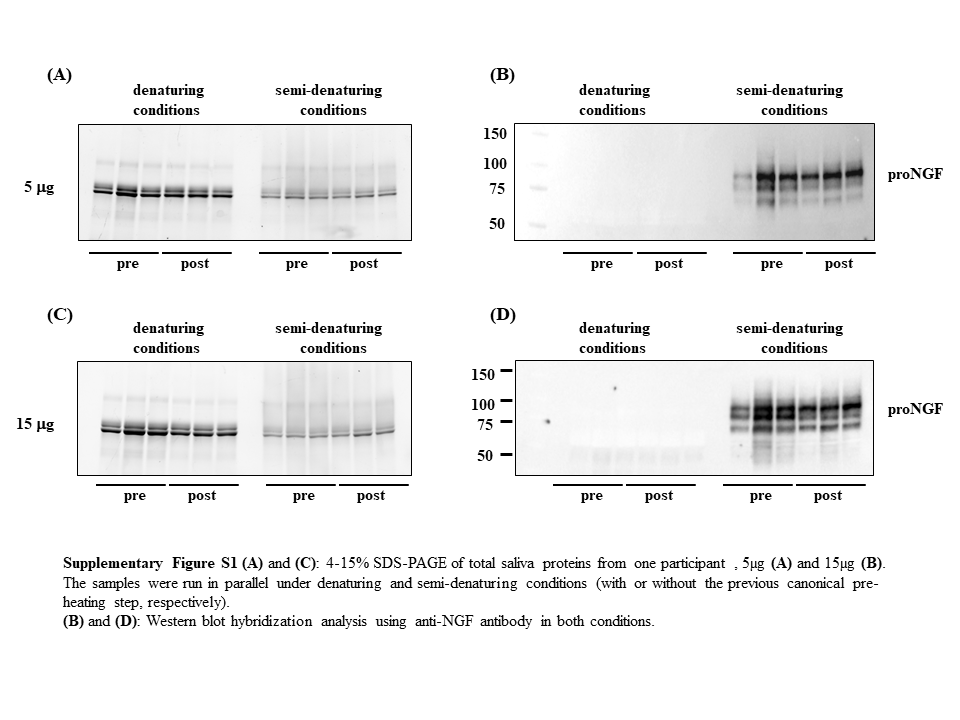

Supplement: Supplementary file 1 [file Image_1.TIF]

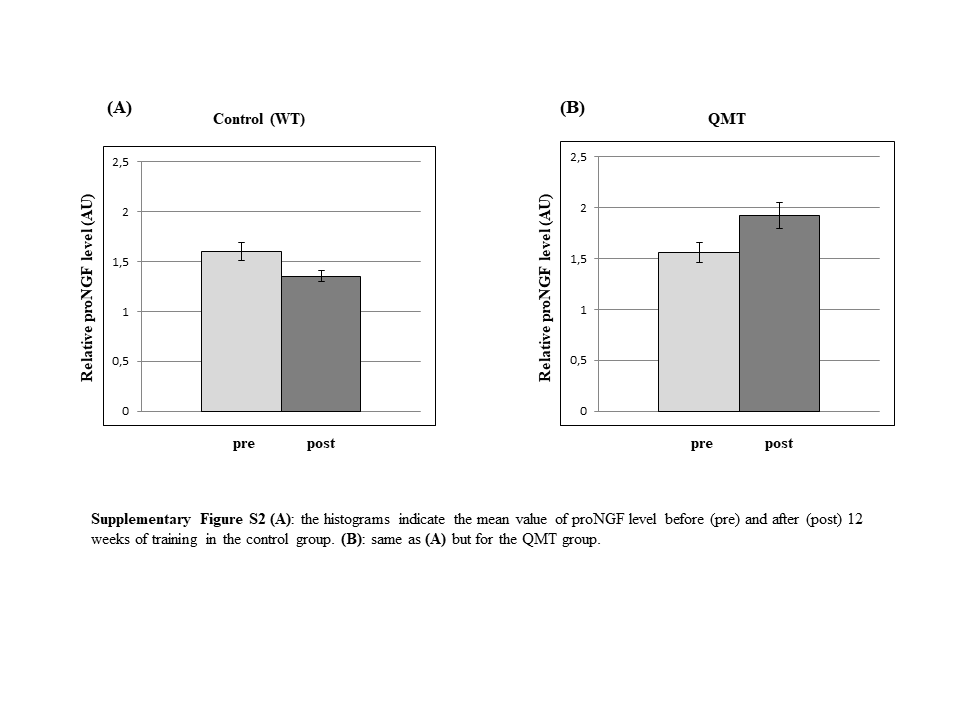

Supplement: Supplementary file 2 [file Image_2.TIF]

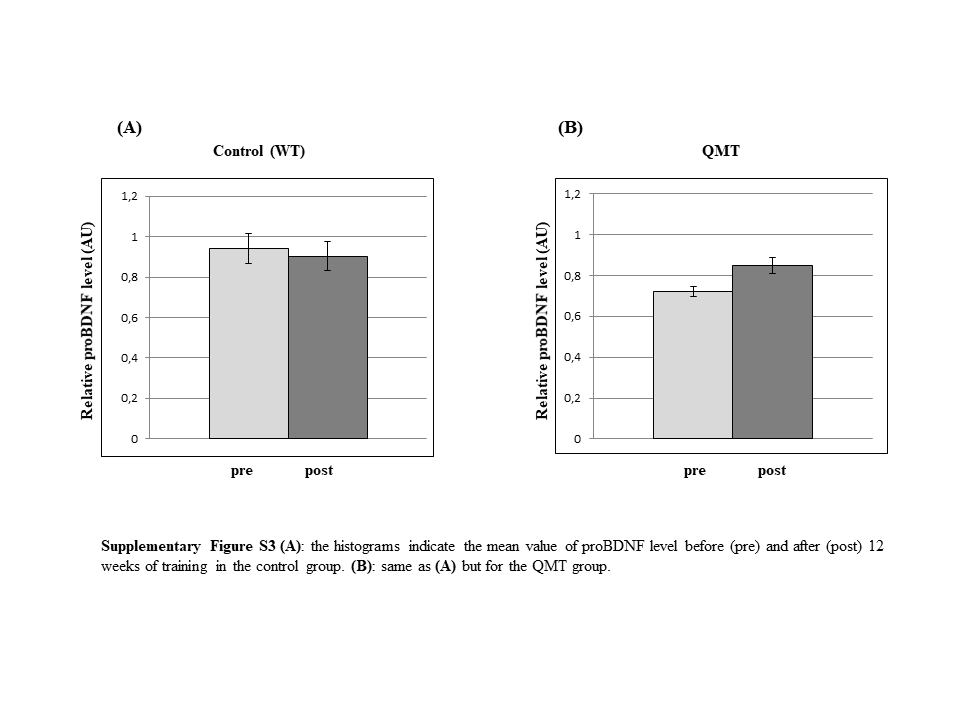

Supplement: Supplementary file 3 [file Image_3.TIF]
